# Gene Copy Number and Post-Transductional Mechanisms Regulate TRAP1 Expression in Human Colorectal Carcinomas

**DOI:** 10.3390/ijms21010145

**Published:** 2019-12-24

**Authors:** Michele Pietrafesa, Francesca Maddalena, Luciana Possidente, Valentina Condelli, Pietro Zoppoli, Valeria Li Bergolis, Maria Grazia Rodriquenz, Michele Aieta, Giulia Vita, Franca Esposito, Matteo Landriscina

**Affiliations:** 1 Laboratory of Preclinical and Translational Research, IRCCS, Referral Cancer Center of Basilicata, Via Padre Pio 1, 85028 Rionero in Vulture, Italy; michele.pietrafesa@crob.it (M.P.); francesca.maddalena@crob.it (F.M.); valentina.condelli@crob.it (V.C.); pietro.zoppoli@crob.it (P.Z.); 2 Pathology Unit, IRCCS, Referral Cancer Center of Basilicata, Via Padre Pio 1, 85028 Rionero in Vulture, Italy; luciana.possidente@crob.it (L.P.); giulia.vita@crob.it (G.V.); 3 Medical Oncology Unit, Department of Medical and Surgical Sciences, University of Foggia, Viale Pinto 1, 71100 Foggia, Italy; valeria.libergolis@unifg.it; 4 Medical Oncology Unit, IRCCS, Referral Cancer Center of Basilicata, Via Padre Pio 1, 85028 Rionero in Vulture, Italy; grazia.rodriquenz@gmail.com (M.G.R.); aietamichele83@gmail.com (M.A.); 5 Department of Molecular Medicine and Medical Biotechnology, University of Naples Federico II, Via Pansini 5, 80131 Naples, Italy

**Keywords:** TRAP1, colorectal carcinoma, copy number variation, GSNOR, S-nitrosylation

## Abstract

Tumor Necrosis Factor Receptor-Associated Protein 1 (TRAP1) is a heat shock protein 90 (HSP90) molecular chaperone overexpressed in 60–70% human colorectal carcinomas (CRCs) and the co-upregulation of TRAP1 and associated 6-related proteins identifies metastatic CRCs with poor prognosis. Since the molecular mechanisms responsible for TRAP1 regulation are still unknown, the significance of *TRAP1* gene copy number (CN) and the role of post-transductional protein modifications were addressed. *TRAP1* gene aneuploidy accounted for 34.5% of cases in a cohort of 58 human CRCs and *TRAP1* CN correlated with its mRNA and protein expression, suggesting that transcriptional mechanisms are responsible for TRAP1 upregulation. Furthermore, the analysis of the National Cancer Institute’s Clinical Proteomic Tumor Analysis Consortium/The Cancer Genome Atlas (CPTAC/TCGA) CRC database showed that *TRAP1* polysomy significantly correlates with lymph node involvement. However, a subgroup of tumors showed TRAP1 protein levels independent from its CN. Of note, a direct correlation was observed between TRAP1 protein levels and the expression of S-nitrosoglutathione reductase (GSNOR), a denitrosylase involved in the regulation of protein S-nitrosylation. Furthermore, CRC cell lines exposed to hypoxia or dichloroacetate treatment showed the downregulation of TRAP1 upon GSNOR silencing and this resulted in increased TRAP1 mono/polyubiquitination. These data suggest that transcriptional and post-transductional mechanisms account for TRAP1 expression in human CRCs and GSNOR protects TRAP1 from S-nitrosylation and consequent proteasome degradation mostly in conditions of stress.

## 1. Introduction

Colorectal carcinoma (CRC) is one of the leading causes of cancer-related morbidity and mortality worldwide [1]. Based on the evidence that CRC is a heterogeneous disease [2], a major aim in this research field is to characterize colorectal tumor biology and define, at the molecular level, tumor subtypes with different prognosis and defined response to therapies [3]. Besides these efforts, at present, few molecular biomarkers have been validated and are available in clinics to predict prognosis and response/resistance to pharmacological agents [4]. Thus, novel biomarkers and/or gene signatures are needed to better predict CRC biological and clinical behavior and select patients for personalized treatments.

Tumor Necrosis Factor Receptor-Associated Protein 1 (TRAP1) is a heat shock protein 90 (HSP90) molecular chaperone, with a prevalent mitochondrial localization, involved in colorectal carcinogenesis, being overexpressed at the transition between low- and high-grade adenomas and in about 60–70% of human CRCs [5]. Indeed, TRAP1 is co-upregulated in the majority of human CRCs with its network of client/related proteins and, through them, regulates several key functions of cancer cells and, among others, adaptation to stress and protection against apoptosis and cytotoxic agents (i.e., oxaliplatin and irinotecan) [6,7], cell cycle progression [8], bioenergetics [9,10,11], and stemness [12]. From a clinical perspective, TRAP1 and its protein network may provide diagnostic/prognostic tools in human CRCs. Interestingly, TRAP1 protein expression correlates with advanced pathologic T-stage [13], extensive lymph node dissemination [14] and, together with high excision repair cross-complementation group 1 (ERCC1), with poor overall survival in metastatic disease [15]. Furthermore, we recently reported a TRAP1 signature based on the co-upregulation of TRAP1 and associated 6-client/related proteins (i.e., eukaryotic initiation factor 2 subunit Alpha (IF2α), eukaryotic translation elongation factor 1 Alpha (eF1A), proteasome regulatory particle TBP7/Rpt3 (TBP7), mitotic arrest deficient 2 (MAD2), cyclin-dependent kinase 1 (CDK1) and β-Catenin) that identifies a cohort of metastatic CRCs with a significantly shorter overall survival [5]. In such a context, a major issue is the understanding of the molecular mechanism responsible for TRAP1 upregulation in human malignancies. The analysis of The Cancer Genome Atlas (TCGA) database showed that the majority of human CRCs are characterized by a diploid *TRAP1* genotype, with a subgroup characterized by gain or loss in *TRAP1* copy number (CN) [5], this suggesting that TRAP1 expression may depend on transcriptional mechanisms. Conversely, recent evidence suggests that post-transcriptional S-nitrosylasion and acetylation mechanisms are responsible for TRAP1 modulation in, respectively, hepatocellular carcinoma and glioma cells [16,17]. Thus, this study was designed to address whether TRAP1 expression in CRC depends on gene CN variation and/or post-transductional mechanisms.

## 2. Results

### 2.1. TRAP1 Expression Is Partially Dependent on Transcriptional Mechanisms

In order to establish whether TRAP1 expression in human CRCs depends on gene CN variation, *TRAP1* CN was analyzed in a cohort of 58 human CRCs at different Tumor, Nodes, Metastasis (TNM) stages (Table 1). 

**Figure 1:**
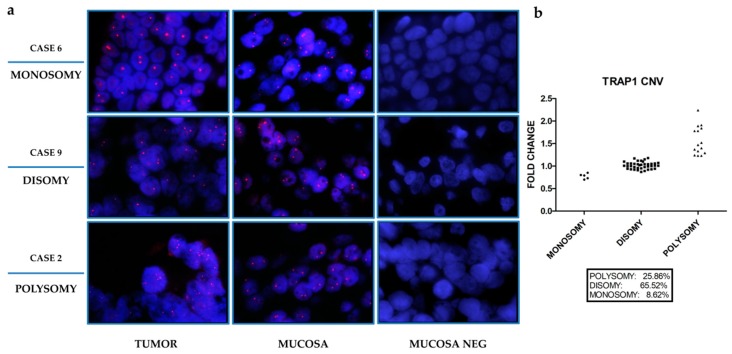
Tumor Necrosis Factor Receptor-Associated Protein 1 (TRAP1) copy number variation in human colorectal carcinomas. (**a**) Fluorescent in situ hybridization (FISH) assessment of *TRAP1* copy number variation in three representative cases of human colorectal carcinoma and the corresponding non-infiltrated mucosa (magnification, 100×). Positive signals of hybridizations are shown in red, nuclei are counterstained with DAPI (Blue). Mucosa lacking *TRAP1* probe (MUCOSA NEG) is reported as a negative control. (**b**) Frequency of *TRAP1* copy number variation evaluated by RT-PCR in 58 human colorectal carcinomas.

To establish cut-offs of *TRAP1* ploidy according to RT-PCR, in preliminary experiments *TRAP1* CN was comparatively evaluated in 15 selected tumors by fluorescent in situ hybridization (FISH) (Figure 1a and Table 2) and RT-PCR (Table 2). 

**Figure 2:**
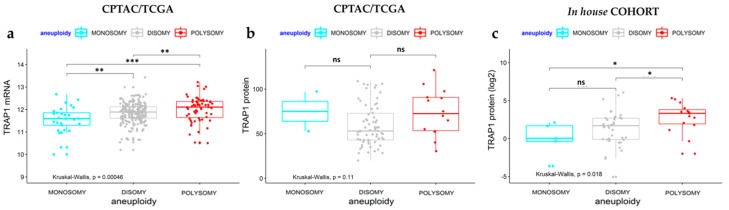
Correlation between *TRAP1* mRNA and protein expression and its copy number variation. (**a**,**b**) Distribution of TRAP1 mRNA (**a**) and protein (**b**) expression according to its copy number variation in the National Cancer Institute’s Clinical Proteomic Tumor Analysis Consortium /The Cancer Genome Atlas (CPTAC/TCGA) colorectal carcinoma database. (**c**) Distribution of TRAP1 protein expression according to its copy number variation in our cohort of human colorectal carcinomas. a. 299 cases b. 81 cases, c. 58 cases. TRAP1 mRNA and protein expression are reported as absolute values in CPTAC/TCGA samples analyzed by RNAseq and mass spectroscopy technologies (**a**,**b**) and as fold change increase respect to non-infiltrated normal mucosa in our in-house cohort analyzed by immunoblot (**c**). *p*-values: * *p* < 0.05, ** *p* < 0.01, *** *p* < 0.001. n.s.: Not significant.

Indeed, *TRAP1* gene CN was considered polysomic for fold changes ≥1.23 and monosomic for fold changes ≤0.85 (Table 2). Based on these cut-off values, *TRAP1* CN was further assessed in the whole cohort of 58 human CRCs by RT-PCR. Indeed, *TRAP1* gene was polysomic in 25.9% of cases, whereas it was monosomic in 8.6% of cases, being the majority of human CRCs (65.5% of cases) disomic (Figure 1b and Supplementary Table S1). Since these data resemble previous results obtained in the TCGA CRC database [5], the correlation between *TRAP1* CN variation and its mRNA and protein expression was further studied in samples from the National Cancer Institute’s Clinical Proteomic Tumor Analysis Consortium (CPTAC) and TCGA database which allowed the analysis of a larger cohort of 539 CRCs (Figure 2a,b).

Interestingly, a progressive increase in *TRAP1* mRNA levels from monosomic to disomic and polysomic tumors was observed (Kruskal–Wallis, *p* = 0.00046; Figure 2a). Consistently, human CRCs with *TRAP1* polysomy were characterized by borderline statistically significant higher protein levels compared to disomic tumors (*p* = 0.053; Figure 2b). No meaningful conclusions can be drawn for monosomic cancers since only two cases with proteomic data are available in CPTAC/TCGA database (Figure 2b). Noteworthy, TRAP1 immunoblot analysis of the cohort of 58 human CRCs showed that tumors with *TRAP1* polysomy are characterized by significantly higher levels of TRAP1 protein compared to disomic or monosomic tumors (Kruskal–Wallis, *p* = 0.0018; Figure 2c and Table S1). No statistical difference was observed between CRCs with *TRAP1* monosomy and disomy (Figure 2c and Table S1).

### 2.2. TRAP1 Polysomy Correlates with N Stage

**Figure 3:**
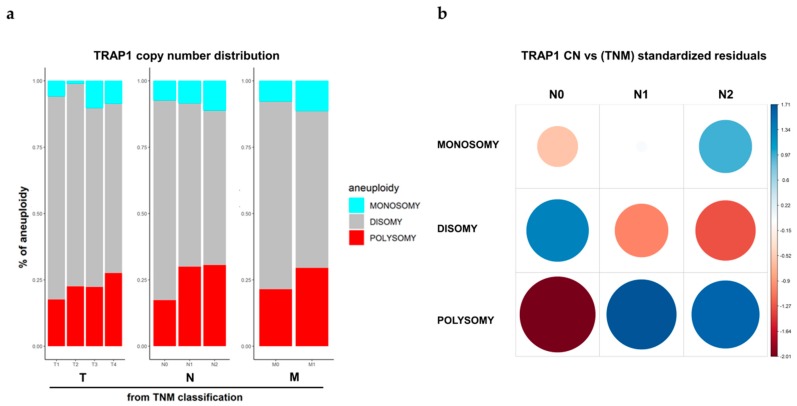
*TRAP1* copy number variation according to the Tumor, Nodes, Metastasis (TNM) stage. (**a**) *TRAP1* copy number distribution according to T, N, and M categories in colorectal carcinomas from TCGA database. (**b**) Corrplot reporting *TRAP1* copy number distribution according to N categories in colorectal carcinomas from TCGA database. The size of the circle is proportional to standardized residuals (statistical significance).

The clinical significance of *TRAP1* CN was explored in TCGA colon and rectum adenocarcinoma (COADREAD) database. In this context, the correlation between *TRAP1* gene CN and T, N, and M categories was studied. Interestingly, we observed a progressive increase in the percentage of tumors with *TRAP1* polysomy according to T, N, and M categories (Figure 3a) and a statistically significant association between *TRAP1* CN and lymph node involvement (Chi-square, *p* = 0.0039), being the frequency of polysomic samples higher in N1 and N2 than in N0 CRCs (Figure 3b). No correlation was observed between *TRAP1* monosomy and TNM stage (Figure 3a).

### 2.3. GSNOR Is Responsible for TRAP1 Post-Transductional Regulation

**Figure 4:**
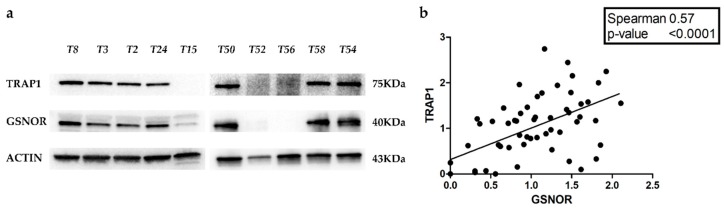
Correlation between TRAP1 and GSNOR protein expression in human colorectal carcinomas. (**a**) TRAP1 and S-nitrosoglutathione reductase (GSNOR) immunoblot analysis in 10 representative cases of human colorectal carcinomas. (**b**) Correlation plot reporting TRAP1 and GSNOR protein expression in 58 human colorectal carcinomas from our cohort (**b**).

These data suggest that transcriptional mechanisms are responsible for TRAP1 protein upregulation, at least in the majority of polysomic tumors, but also in several disomic malignancies. However, there is a subgroup of human CRCs characterized by TRAP1 protein upregulation independently from gene CN variation and mRNA expression, suggesting that post-transductional mechanisms are also likely to play a role in regulating TRAP1 expression in colorectal tumors. From such a perspective, recent evidence suggests that TRAP1 protein stability is controlled by S-nytrosilation and that this process is mediated by S-nitrosoglutathione reductase (GSNOR), a denitrosylase implicated in regulating the levels of proteins post-translationally modified by S-nitrosylation [16]. Thus, GSNOR protein levels were evaluated, by immunoblot analysis, in our cohort of human 58 CRCs and correlated with TRAP1 protein expression (Figure 4a). Of note, Spearman correlation test showed a positive correlation between TRAP1 and GSNOR protein levels (R = 0.57, *p* < 0.0001) (Figure 4b).

**Figure 5:**
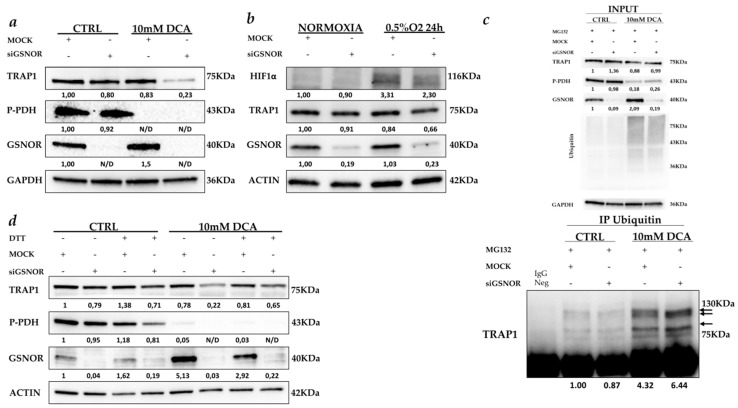
GSNOR protects TRAP1 from S-nitrosylation. (**a**) TRAP1, phospho-pyruvate dehydrogenase (P-PDH) and GSNOR immunoblot analysis in Mock- and GSNOR-silenced HCT116 cells cultured in the presence and the absence of 10 mM dichloroacetate (DCA) for 24 h. (**b**) TRAP1, Hypoxia-inducible factor 1-alpha (HIF1α) and GSNOR immunoblot analysis in Mock- and GSNOR-silenced HCT116 cells cultured in normal conditions (normoxia) or in presence of 0.5% O_2_ (hypoxia) for 24 h. (**c**) TRAP1 immunoblot analysis of anti-ubiquitin immunoprecipitates from Mock- and GSNOR-silenced HCT116 cells cultured in the presence and the absence of 10 mM DCA for 24 h. Cell lines were incubated with 10 µM Mg132 for 4 h before cell harvesting. Arrows indicate bands of TRAP1 mono/polyubiquitination. Input: TRAP1, P-PDH GSNOR and ubiquitin immunoblot analysis in Mock- and GSNOR-silenced HCT116 cells cultured as indicated in c. (**d**) TRAP1, P-PDH, and GSNOR immunoblot analysis in Mock- and GSNOR-silenced HCT116 cells cultured in the presence and the absence of 10 mM DCA for 24 h and further incubated with 0.5 mM dithiothreitol (DTT) for 3 h before cell harvesting. N/D, not detectable.

These data support the hypothesis that S-nitrosylation is involved in the regulation of TRAP1 stability/degradation in colorectal tumors. To establish the role of GSNOR in protecting TRAP1 from S-nitrosylation and consequent degradation, we generated a model of GSNOR silencing in CRC cells exposed to stress conditions. Mock- and GSNOR-silenced CRC HCT116 cells were incubated for 24 h in presence of 10 mM dichloroacetate (DCA) (Figure 5a), an agent that induces loss of pyruvate dehydrogenase (PDH) phosphorylation with parallel enhancement of oxidative metabolism, production of nitric oxide (NO) and consequent protein nitrosylation [18] or under hypoxia (Figure 5b), a condition that results in increased NO production [19]. TRAP1 protein level was evaluated, by immunoblot analysis, to assess the hypothesis that GSNOR knock out favors its downregulation under stress condition. 

In parallel experiments, both 10 mM DCA treatment and hypoxia were shown to not induce apoptotic cell death in HCT116 cells [20]. Interestingly, TRAP1 levels were almost unchanged in stressed cells with conserved expression of GSNOR or upon its silencing in the absence of stress (Figure 5a,b). By contrast, GSNOR silencing in HCT116 cells exposed to DCA (Figure 5a) or hypoxia (Figure 5b) resulted in a significant TRAP1 protein downregulation, which is more evident upon DCA treatment. Importantly, *TRAP1* mRNA levels were unchanged in the same experimental conditions [20], confirming that TRAP1 downregulation is mediated by post-transductional mechanisms. 

Since Rizza et al. recently reported that TRAP1 S-nitrosylation favors protein ubiquitination and subsequent proteasome degradation [16], TRAP1 ubiquitination was evaluated in stress conditions and upon GSNOR silencing. Thus, ubiquitinated proteins were immunoprecipitated from Mock- and GSNOR-silenced cells exposed to DCA for 24 h (Figure 5c). In these experiments, protein degradation was partially prevented by incubation of cells with the proteasome inhibitor, MG132 as documented by increased levels of protein ubiquitination under DCA treatments (Figure 5c, upper panel). Noteworthy, TRAP1 immunoblot analysis showed increased levels of TRAP1 mono/polyubiquitination under DCA exposure, and this was further increased upon GSNOR silencing (Figure 5c, lower panel). To address the hypothesis that TRAP1 protein S-nitrosylation is the main mechanism responsible for its downregulation under stress conditions, S-nitrosylation was inhibited, upon incubation with dithiothreitol (DTT), in GSNOR-silenced HCT116 cells exposed to DCA (Figure 5d). Noteworthy, DTT partially rescued TRAP1 downregulation despite GSNOR silencing and exposure to DCA in HCT116 cells (Figure 5d). Altogether, these data suggest that TRAP1 is regulated by posttranslational modifications under stress conditions and that GSNOR protects TRAP1 from S-nitrosylation and ubiquitin degradation.

## 3. Discussion

Several genes have been described to act as oncogenes or oncospuppresors in a context- and tumor-dependent manner [21,22,23,24]. In the case of TRAP1, the majority of human malignancies are characterized by its upregulation (i.e., colorectal, lung, breast and prostate carcinomas), whereas selected tumors by its downregulation (i.e., renal, cervical and ovarian carcinomas) [25]. In this scenario, TRAP1 acts as an oncogene or oncosuppressor gene depending on tumor type [26] and cancer cells up/downregulate TRAP1 expression to adapt to unfavorable environments and remodel cell bioenergetics to fulfill high-energy demanding conditions [26]. Indeed, TRAP1 is responsible for co-translational quality control of a network of client/related proteins and, through them, regulates several cell functions (i.e., apoptotic signaling, cell metabolism, cell cycle progression, and stemness) [8,12,27,28]. Thus, the modulation of its expression may provide cancer cells with a mechanism to rapidly modify specific signaling pathways and adapt to environmental changes. In the context of human CRC, TRAP1 acts as an oncogene [6,12,13,29,30] and its upregulation is responsible for driving tumor progression and patients’ prognosis [5,31,32].

In this study, the mechanism of TRAP1 modulation in colorectal carcinoma was addressed in in vivo and in vitro models. Our data suggest that a subgroup of human CRCs is characterized by gain or loss in *TRAP1* gene CN and that there is a direct correlation between *TRAP1* CN and its mRNA and protein expression, suggesting a transcriptional regulation for this molecular chaperone. However, there is a considerable number of cases, mostly tumors with a monosomic/disomic *TRAP1* genotype, that exhibit a wide distribution of its expression independently from CN variation and mRNA expression, suggesting that post-transductional modifications may play a pivotal role in TRAP1 protein stability/degradation. Indeed, post-transductional S-nitrosylation modifications are likely to be involved in favoring TRAP1 degradation by the proteasome especially under stress conditions. Two pieces of evidence support this hypothesis: i) The statistically significant correlation between TRAP1 protein levels and the expression of GSNOR in human CRCs and ii) the GSNOR protecting activity toward TRAP1 ubiquitin degradation in stress conditions in vitro. 

To our knowledge, this is the first study, which addresses the role of *TRAP1* CN variation in human CRCs, showing that transcriptional mechanisms drive TRAP1 upregulation in human colorectal malignancies. A similar observation was previously obtained by our group in human ovarian carcinoma, a malignancy characterized by TRAP1 downregulation with parallel loss of its gene CN across tumor stage and development of platinum resistance [28,33]. This conclusion is supported by the higher levels of *TRAP1* mRNA mostly in tumors with gain in *TRAP1* CN and by the significant correlation between *TRAP1* CN and *TRAP1* mRNA and protein expression (this study and [5]), suggesting the relevance of transcriptional mechanisms in driving TRAP1 upregulation in selected colorectal malignancies. 

It is noteworthy that a relevant number of human CRCs is characterized by high TRAP1 protein levels independently from gene CN variation, and this mostly occurs in cases with *TRAP1* monosomic/disomic genotype. In these tumors, it is likely that TRAP1 stability is regulated by post-transductional modifications and that denitrosylation mechanisms, driven by GSNOR activity, are likely to protect TRAP1 from S-nitrosylation and consequent proteasome degradation. This hypothesis is consistent with recent evidence showing that aberrant S-nitrosylation in hepatocellular carcinoma, due to GSNOR deficiency, results in mitochondrial alteration and parallel upregulation of succinate dehydrogenase levels and activity, this depending on TRAP1 S-nitrosylation and subsequent degradation [16]. Furthermore, it has been recently proposed that TRAP1 is acetylated upon interaction with sirtuin-3 in mitochondria of glioma cells [17]. Indeed, our in vitro data suggest that this mechanism is particularly relevant in response to environmental stress, thus favoring cancer cell protection from unfavorable conditions and driving survival adaptive mechanisms. Thus, it is intriguing to speculate that TRAP1 stability is regulated by post-transductional modifications especially under stress conditions and this allows cancer cells to rapidly modify TRAP1 expression and its downstream protein network in response to extracellular stimuli. In such a context, GSNOR is likely to protect TRAP1 from S-nitrosylation and consequent proteasome degradation. This hypothesis is consistent with the established role of TRAP1 as heath shock protein [25,26] and its involvement in protection from oxidative and endoplasmic reticulum stress, activation of antiapoptotic pathways, drug resistance and metabolic rewiring [6,9,11,34,35,36]. It is important to note that our data do not allow excluding that other molecular mechanisms, besides GSNOR and protein S-nitrosylation, are involved in the regulation of TRAP1 stability, which represents a field of further investigation.

Finally, this study provides relevant information in the perspective to validate TRAP1 and its protein network as prognostic/predictive biomarkers in human CRCs. Indeed, *TRAP1* gene CN may represent a surrogate marker of its expression in colorectal tumors and correlate with lymph node involvement in a large cohort of human CRCs and thus deserves to be evaluated as a reliable diagnostic/prognostic tool for clinical use. However, since post-transductional mechanisms are also involved in regulating TRAP1 stability in a relevant subgroup of human CRCs, this suggests that the characterization of TRAP1 protein expression, together with the evaluation of its protein network, may represent a more appropriate methodology to further establish its clinical significance. 

In conclusion, this study supports the concept that either transcriptional or transductional mechanisms are responsible for TRAP1 regulation in human CRCs. Thus, TRAP1 protein evaluation is the most reliable tool to assess its expression in human CRC samples. 

## 4. Materials and Methods

### 4.1. Tumor Specimens, Clinical Data, Reagents and Cell Cultures

A cohort of 58 human CRCs was obtained from the IRCCS-CROB Tissue Biobank. Tumor specimens and the corresponding normal, non-infiltrated peritumoral mucosa were obtained after surgical removal and immediately frozen in liquid nitrogen. Tumors were staged according to TNM classification system [37]. Patient characteristics are reported in Table 1. All patients gave their informed written consent to use biological specimens for investigational procedures. The study was approved by the local Ethics Committee (Registration number: A608051, date of approval: 2012-08-05).

A cohort of 616 gene-level CN variation, 434 RNAseq and 736 clinical annotations samples were retrieved from TCGA (https://www.cancer.gov/tcga) COADREAD. Ninety proteomic data samples were downloaded from CPTAC (https://proteomics.cancer.gov/programs/cptac). Overlapping samples were used for the analysis (539 cases for the correlation between *TRAP1* CN variation and clinical annotations, 299 cases for the correlation between *TRAP1* CN variation and mRNA expression and 81 cases for correlation between CN variation and protein expression).

Human CRC HCT116 cells were purchased by the American Type Culture Collection (ATCC, Manassas, VA, USA) and grown in McCoy’s supplemented with 10% FBS, L-glutammine 2 mM and antibiotics (penicillin-streptomycin 100 U/mL) at 37 °C in an atmosphere of 5% CO2. In specific experiments, cell lines were placed in a humidified O_2_-control incubator (Galaxy 48R, New Brunswick, Eppendorf, Hamburg, Germany) at 37 °C, 0.5% O_2_ for 24 h. At the same time, normoxia cells were placed at 37 °C in a 20% O_2_ and 5% CO_2_ incubator.

Cells were routinely monitored in our laboratory by microscopic morphology and for mycoplasma detection, whereas their authentication was verified by STR profiling, according to the ATCC product description. All tissue culture reagents were purchased from Gibco, Thermo Fisher Scientific, Inc., (Waltham, MA, USA). siRNAs for GSNOR were purchased from Qiagen (Hilden, Germany, cat. No. SI03047660), diluted to a final concentration of 20 nmol/L and transfected by hiperfect trasfection reagent according to the manufacturer′s protocol. For control experiments, cells were transfected with a similar amount of scrambled siRNA (Qiagen, Hilden, Germany, Cat. No.SI03650318). DCA (Sigma-Aldrich, Saint Louis, MO, USA) was used at a final concentration of 10 mM. DTT (Sigma-Aldrich, Saint Louis, MO, USA) was used for 3 h at a final concentration of 0.5 mM.

### 4.2. Cell Extract, Immunoblot Analysis, and Antibodies

Immunoblot analysis, obtained by homogenization of cell pellets and tissue samples, was done as previously reported [38,39]. Protein immunoprecipitation was performed starting from 1 mg of total proteins by Pierce Classic IP kit (Thermo Scientific, Inc.), according to the manufacturer’s protocol. Lysates were incubated with gentle shaking for 18 h at 4 °C with specific antibodies. The following antibodies were used: Mouse monoclonal anti-HSP75 (sc-73604), mouse monoclonal anti-ubiquitin (sc-8017), mouse monoclonal anti-GAPDH (sc-47724) and mouse monoclonal anti-β-Actin (sc-47778) from Santa Cruz Biotechnology (Dallas, TX, USA), rabbit polyclonal anti-ADH5 (GSNOR) (16379-1-AP) from Proteintech (Rosemont, IL, USA), rabbit polyclonal phospho-PDH-E1α from Abcam (Cambridge, UK). Protein levels were quantified by densitometric analysis using the ImageJ software and normalized according to the expression of the housekeeping gene and compared to normal mucosa.

### 4.3. RT-PCR Copy Number Variation Analysis

Total DNA was extracted from 10µg specimens of tumors and corresponding normal mucosa using Allprep DNA\RNA mini kit (Qiagen) according to manufacturer’s protocol. A pre-designed Taqman CN Assay was chosen to detect *TRAP1* CN, (Applied Biosystems, Thermo Scientific, Inc. Cat. No.Hs02824034_cn) mapping the exon 10 (FAM dye-labeled). RNASEP (VIC dye-labeled) (Applied Biosystems, Thermo Scientific, Inc. Cat. No.4403328) was used as a reference gene. To evaluate the efficiency of the assay, a standard curve was generated, using DNA extracted from PMBCs of three healthy individuals, making a serial dilution 1:2, 1:1, 1.5:1, 2:1 of the sample running with the *TRAP1* probe. The same sample running with the RNASEP probe was used with no dilution in a separate reaction. DNA extracted from PMBCs of a patient with a lymphoproliferative disorder bearing monosomy of chromosome 16 was used as control. For RT-PCR analysis, 10 ng of DNA samples were amplified using the Taqman Genotyping Master mix (Applied Biosystems, Thermo Scientific, Inc.) in a LightCycler 480 (Roche). PCR reaction conditions were as follows: Pre-incubation at 95 °C for 30 s, followed by 45 cycles of 5 s at 95 °C, 30 s at 60 °C. Each specimen was analyzed in triplicate and quantified with the 2-ddct method.

### 4.4. Fluorescent In Situ Hybridization (FISH)

FISH analysis for *TRAP1* gene was evaluated on 4 μm thick tissue sections. Specimens were formalin-fixed and paraffin-embedded (FFPE), according to the manufacturer’s instructions (DakoCytomation, Glostrup, Denmark). FFPE tissue sections were placed at 60 °C for 60 min, deparaffinized and rehydrated. Samples were incubated in a pretreatment solution for 10 min at 95 °C and digested with pepsin solution for 6 min at 37 °C. The probe specific for *TRAP1* locus on chromosome 16 was custom designed by Agilent Manufacturing and hybridized according to the manufacturer’s protocol. Post-hybridization stringency wash was carried out in water bath at 65 °C for 10 min. After washing twice and drying at room temperature for 15 min, slides were mounted with fluorescent mounting medium containing 4′6-diamidine-2-phenylindole (DAPI, DakoCytomation, Glostrup, Denmark). FISH signals were evaluated with Nikon Eclipse 80i with single and triple band pass filters. The acquisition of images was processed using the Genikon System. At least 100 tumor cells were scored for the analysis of CN signals. Tumor was considered monosomic for *TRAP1* gene if >50% of cells showed one copy, disomic if >90% of cells showed ≤2 copies, polysomic if at least 50% of cells showed >2 copies [40,41].

### 4.5. High-Throughput Sequencing and Other Statistical Analyses

Kruskal–Wallis test was used to establish statistical differences in *TRAP1* expression between CN variation and its mRNA and protein expression in human CRC specimens in both our cohort of 58 human CRCs and TCGA dataset. To investigate the association between *TRAP1* CN and the degree of intestinal wall invasion and the spread to regional lymph nodes and distant organs (respectively, T, N, and M categories of TNM staging system) a Chi-square test was performed. In order to pinpoint the most contributing sample group to the total Chi-square score, we calculated the Pearson residuals (r) for each of them (or standardized residuals) and plotted a correlation plot (corrplot) where, for a given sample group, the size of the circle is proportional to the amount of the sample group contribution (blue positive while red negative association between *TRAP1* CN and N). Spearman rank-order correlation method was used to calculate the correlation coefficient and the p-value between TRAP1 and GSNOR protein expression in human CRC samples. Statistically significant values are reported in figures and text. All the analyses and the plots were performed using R [42] and survival, ggplot2, ggpubr, and corrplot packages [43,44,45,46]. All experiments were independently performed at least three times, and three technical replicates were used for statistical analysis. Data represent means ± S.D.

## Figures and Tables

**Table 1:** Patients’ baseline characteristics.

Patients	58	
**Age**		
***Median (years)***	70	
***Range***	35–89	
**Sex**	No of patients	
***Female***	26	
***Male***	32	
**Tumor stage**	No of patients	(%)
***Tumor***		
***T1***	2	4
***T2***	4	7
***T3***	38	65
***T4***	14	24
***Nodes***		
***N0***	19	33
***N1***	19	33
***N2***	20	34
***Metastasis***		
***M0***	38	65
***M1***	20	35

**Table 2:** Comparative fluorescent in situ hybridization (FISH) and RT-PCR analysis of *TRAP1* gene CN.

Cases (*n*)	*TRAP1* Copy Number for Cells (%)			Pattern	Fold Change
	1 Signals/Cell	2 Signals/Cell	>2 Signals/Cell		
**1**			100	Polisomy	1.84
**2**		3	97	Polisomy	1.24
**3**		4	96	Polisomy	1.23
**4**	63	31	6	Monosomy	0.73
**5**	16	80	4	Disomy	1.05
**6**	63	37		Monosomy	0.85
**7**	17	29	54	Polisomy	1.52
**8**	21	72	7	Disomy	1.12
**9**	41	56	3	Disomy	0.91
**10**	12	82	6	Disomy	1.17
**11**	15	80	5	Disomy	1.06
**12**	10	82	8	Disomy	1.03
**13**	17	75	8	Disomy	1.1
**14**	15	79	6	Disomy	1.04
**15**	38	60	2	Disomy	0.94

## Supplementary Materials

The following are available online at https://www.mdpi.com/1422-0067/21/1/145/s1.

## Abbreviations

TRAP1	Tumor Necrosis Factor Receptor-Associated Protein 1
TCGA	The Cancer Genome Atlas
CPTAC	the National Cancer Institute’s Clinical Proteomic Tumor Analysis Consortium
CRCs	Human Colorectal Carcinomas
CN	Copy number
COADREAD	Colon and rectum adenocarcinoma
GSNOR	S-nitrosoglutathione reductase
DCA	Dichloroacetate
PDH	pyruvate dehydrogenase
DTT	dithiothreitol
