# Supplementary material for: Gene Copy Number and Post-Transductional Mechanisms Regulate TRAP1 Expression in Human Colorectal Carcinomas
Source: Int J Mol Sci. 2019 Dec 24;21(1):145. doi: 10.3390/ijms21010145 (PMC6981705; doi:10.3390/ijms21010145)
Supplement: Supplementary file 1 [file ijms-21-00145-s001.pdf]

The data used to support the findings of this study are included within the article with the exception of the data regarding the correlation between TRAP1 copy number and its protein expression in human CRCs that are included within the supplementary information file and the data regarding the correlation between TRAP1 and GSNOR expression in human CRCs that are available from the corresponding author upon request.

Supplementary Table S1. Trap1 expression in human colorectal cancers.

CASES (n.)	CNV	PATTERN	PROTEIN	CASES (n.)	CNV	PATTERN	PROTEIN
**1**	1.84	Polisomy	11,55	**30**	0,94	Disomy	2,83
**2**	1.24	Polisomy	26,99	**31**	0,87	Disomy	11,62
**3**	1.23	Polisomy	7,67	**32**	1,23	Polisomy	11,02
**4**	0.73	Monosomy	0,77	**33**	1,4	Polisomy	0,79
**5**	1,05	Disomy	2,18	**34**	1,1	Disomy	8,48
**6**	0.85	Monosomy	0,08	**35**	1,04	Disomy	3,23
**7**	1.52	Polisomy	40,2	**36**	1,78	Polisomy	3,46
**8**	1.12	Disomy	4,05	**37**	1,06	Disomy	2,21
**9**	0.91	Disomy	3,69	**38**	1,78	Polisomy	4,16
**10**	1.17	Disomy	7,38	**39**	1,18	Disomy	1,07
**11**	1,06	Disomy	5,55	**40**	1,15	Disomy	67,59
**12**	1,03	Disomy	4,25	**41**	1,29	Polisomy	12,2
**13**	1,1	Disomy	12,26	**42**	0,96	Disomy	1,1
**14**	1,04	Disomy	0,94	**43**	2,24	Polisomy	0,25
**15**	0,94	Disomy	0,03	**44**	0,98	Disomy	0,98
**16**	1.89	Polisomy	6,78	**45**	1,33	Polisomy	36,42
**17**	1,02	Disomy	0,7	**46**	0,93	Disomy	0,9
**18**	0,92	Disomy	6,49	**47**	0,99	Disomy	0,43
**19**	0,79	Monosomy	1,02	**48**	1,01	Disomy	2,67
**20**	1,12	Disomy	6,77	**49**	0,89	Disomy	36,74
**21**	1,06	Disomy	6,27	**50**	1,47	Polisomy	10
**22**	1,01	Disomy	52,18	**51**	1,01	Disomy	1,33
**23**	0,93	Disomy	3,5	**52**	1,07	Disomy	0,33
**24**	0,8	Monosomy	3,2	**53**	0,95	Disomy	0,91
**25**	0,98	Disomy	0,69	**54**	1,91	Polisomy	16,04
**26**	1,37	Polisomy	6,73	**55**	0,7	Monosomy	4,31
**27**	1,05	Disomy	3,23	**56**	1	Disomy	0,18
**28**	1,04	Disomy	5,73	**57**	1,0	Disomy	0,16
**29**	0,92	Disomy	0,81	**58**	1	Disomy	3,7
